# Changes of gut microbiota in patients at different phases of stroke

**DOI:** 10.1111/cns.14271

**Published:** 2023-06-12

**Authors:** Wei Cui, Li Xu, Lin Huang, Yang Tian, Yan Yang, Yamei Li, Qian Yu

**Affiliations:** ^1^ Department of Rehabilitation Medicine Sichuan Provincial People's Hospital, University of Electronic Science and Technology of China Chengdu China

**Keywords:** 16S rRNA, gut microbiota, rehabilitation, stroke

## Abstract

**Aims:**

Gut dysbiosis appears rapidly after acute stroke and may affect the prognosis, whereas changes in gut microbiota with gradual recovery from stroke are unknown and rarely studied. The purpose of this study is to explore the characteristics of gut microbiota changes over time after stroke.

**Methods:**

Stroke patients and healthy subjects were selected to compare the clinical data and gut microbiota of the patient group in two phases with that of healthy subjects and 16S rRNA gene sequencing was used to search the differences of gut microbiota in subjects.

**Results:**

Compared with the healthy subjects, the subacute patients mainly decreased the abundance of some gut microbial communities, while the decreased communities reduced and more communities increased the abundance in the convalescent patients. The abundance of *Lactobacillaceae* increased in both phases in patient group, while *Butyricimona*, *Peptostreptococaceae* and *Romboutsia* decreased in both phases. Correlation analysis found that the MMSE scores of patients in the two phases had the greatest correlation with the gut microbiota.

**Conclusion:**

Gut dysbiosis still existed in patients in the subacute phase and convalescent phase, and gradually improved with the recovery of stroke. Gut microbiota may affect the prognosis of stroke by affecting BMI and/or related indicators, and there is a strong correlation between gut microbiota and cognitive function after stroke.

## Graphical abstract

Gut dysbiosis still existed in patients in the subacute phase and convalescent phase, and gradually improved with the recovery of stroke; *Lactobacillaceae* increased in both phases compared to the healthy subjects. Gut microbiota may affect the functional status of stroke by affecting BMI and/or cognitive scores, thereby changing the prognosis of stroke.

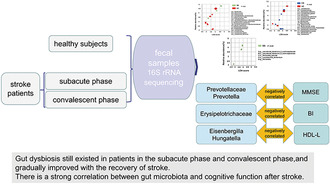

## 1 INTRODUCTION

Stroke is a focal damage of the central nervous system caused by vascular injuries, and often leads to deaths or permanent neurological damages.^1^ Stroke is the main cause of physical disability in adults, resulting in about 44 million physical disabilities every year.^2^, ^3^ Although the medical community has put in great effort to improve the prognosis of post‐stroke patients, many patients still suffer from severe sequelae such as motor dysfunction, cognitive dysfunction, aphasia, and swallowing dysfunction, which imposes a heavy burden on the patient's family and society. Therefore, it is imperative to develop new treatment regimens to improve the clinical outcomes of post‐stroke patients.

The gut microbiota is a group of dynamic, complex and diverse microbial population residing in our gut^4^; it is considered the second genome of human body, making important contributions to our physiology and metabolism, and sharing some vital functions with the host.^5^ The healthy microbiota helps to maintain the integrity of the blood–brain barrier. When the diversity, abundance, and function of microbiota change to a certain extent, the microbiota and its host may no longer be able to restore the symbiotic state, gut dysbiosis occurs.^6^Gut dysbiosis is usually associated with increased intestinal barrier dysfunction and local inflammation, affecting the development and functional regulation of the host's immune, metabolic and nervous system,^7^, ^8^ and could cause various diseases, such as Alzheimer's disease,^9^ Parkinson's disease,^10^ multiple sclerosis, and Guillain‐Barre syndrome among others.^11^ Recent studies have shown that there is a bidirectional communication network between the brain and gastrointestinal (GI) tract. This network, known as the gut–brain axis (GBA), connects the central cognitive and emotional centers of the brain with the peripheral gut, and it involves the gut microbiota, gut, and nervous system.^12^ GBA connects the brain and intestine through direct and indirect pathways, involving the central nervous system, neuroendocrine (hypothalamus‐pituitary–adrenal axis), neuroimmune and autonomic nervous systems (sympathetic, parasympathetic and enteric nervous systems).^12^Gut microbiota affects the enteric nervous system and generate afferent signals from the gut to the brain by stimulating the vagus nerve.^13^ The nervous system can either directly affect the gut and gut microbiota by secreting signal molecules via the neuron cells in the lamina propria, immune cells, and enterochromaffin cells, or indirectly affect the intestinal microenvironment by regulating the movement, secretion and permeability of the GI tract through autonomic nerves.^14^ It has been found that gut microbiota is associated with ischemic stroke along the GBA, and that gut dysbiosis can affect local immune cells in the gut and brain, leading to GBA dysfunction.^8^ In a rat model of stroke, gut dysbiosis exacerbated the transport of T cells from the gut to the central nervous system, leading to chronic and systemic neuroinflammation.^15^

In recent years, the medical community has become increasingly interested in the pathological changes of stroke mediated by gut dysbiosis and potential new treatment regimens of stroke involving the alteration of gut microbiota. Sadler et al^16^ studied the mechanism between acute stroke, microbiota changes and immune response after brain injury, and they discovered that stroke lesions led to gut microbiota imbalance, which influenced the prognosis of stroke through immune‐mediated mechanisms. Tan et al^17^ confirmed that acute ischemic stroke induced gut dysbiosis, and these changes in gut microbiota in turn affected the neuroinflammatory response and stroke. Xu et al^18^ conducted cohort studies on ischemic stroke patients and animal models and further confirmed that rapid gut microbiota dysbiosis caused by stroke accelerated the degree of cerebral infarction. Therefore, we speculate that gut dysbiosis affects neuroinflammation, metabolism and immune homeostasis after brain injury, which in turn influences the progression and prognosis of stroke via immune‐mediated response.

In summary, existing studies have confirmed that gut dysbiosis is related to the onset of stroke, and that the gut microbiota is rapidly dysregulated in acute stroke, which further aggravates the stroke. And the deregulation of GBA and alterations in gut microbial compositions are also noted in the subacute phase of stroke. However, there are few studies on the change of gut microbiota in the subacute phase of stroke and in the convalescent phase after rehabilitation treatment. In this study, the clinical data and gut microbiota status of subacute stroke patients, convalescent stroke patients and healthy people are compared, aiming to uncover the changes of gut microbiota in patients at different phases after stroke, identify potential prognostic biomarkers of stroke, and explore the role of gut microbiota in the recovery of stroke. This study could potentially lay a foundation for future research on the treatment of stroke based on gut microbiota.

## 2 MATERIALS AND METHODS

### 2.1 Study participants

Fourteen stroke patients admitted into the rehabilitation department of Sichuan Provincial People's Hospital from July to September 2021 were selected as the patient group, and 17 healthy subjects in the physical examination center of the hospital during the same period were selected as the healthy control (HC) group. When initially enrolled, all subjects in the patient group were assessed with subacute phase stroke, and the group was named subacute stroke (SS) group. The assessment after 4 weeks showed that all subjects were in the convalescent phase, and hence the group was named convalescent stroke (CS) group.^18^ This study was approved by the Medical Ethics Committee of Sichuan Provincial People's Hospital. Clinical registration number is ChiCTR2100049001.

The inclusion criteria included: (1) The patients met the diagnostic criteria of stroke and were confirmed by brain computed tomography (CT) or magnetic resonance imaging (MRI); (2) The patients had the first‐ever stroke, and the time of inclusion was 7–15 days since the onset of stroke; the healthy controls had no history of stroke; (3) The subjects were aged 40 to 80 years old at the time of inclusion; (4) The subjects had been living in Sichuan Province for more than 3 years for more than 10 months per year; (5) The subjects had no special dietary habits; (6) The subjects did not take any probiotics, prebiotics, and antibiotics within the past month; and (7) Informed consents were signed by the subjects or the authorized principals.

The exclusion criteria included: (1) The patients had brain tissue damages not caused by stroke; the healthy controls had brain trauma or other intracranial diseases; (2) The subjects were suffering from serious digestive system diseases such as inflammatory gastrointestinal diseases, serious autoimmune diseases, and serious liver diseases; (3) The subjects were suffering from serious life‐threatening diseases such as heart failure and respiratory failure; and (4) The subjects were unable to cooperate or suffering from conditions that would interfere with the behavioral assessment and treatment.

### 2.2 Study design

#### 2.2.1 Treatment

All patients received routine clinical treatments, including trophic nerve, improvement of microcirculation, controlling blood pressure, controlling blood glucose and other drug therapy. All patients received rehabilitation treatments at the same time, including physical therapy, occupational therapy, and acupuncture among others.

#### 2.2.2 Evaluation

Mini‐Mental State Examination (MMSE) with scores ranging from 0 to 30 was used to assess the cognitive function of the patients; a higher score indicated better cognitive functions.^19^ The Fugl‐Meyer Assessment (FMA) with a maximum score of 100 was used to assess the degree of motor dysfunction of both upper and lower limbs in patients; a higher score indicated better motor functions.^20^ The Barthel Index (BI) with scores ranging from 0 to 100 was used to assess the ability of daily living activities of patients; a higher score indicated better ability of performing daily living activities.^21^

#### 2.2.3 Fecal samples and clinical data acquisition

Specialized fecal collection boxes and fecal DNA preservation solution were used to collect and preserve fecal samples of all subjects. Demographic and clinical data, including age, sex, type of stroke, time since onset, body mass index (BMI), waist‐hip ratio (WHR), high‐density lipoprotein cholesterol (HDL‐C), low‐density lipoprotein cholesterol (LDL‐C), triglyceride (TG), total cholesterol (TC), uric acid (UA), homocysteine (Hcy), red blood cells (RBC), white blood cells (WBC), platelet count (PLT), total serum protein (TP), serum albumin (Alb) and creatinine (Cre), were collected.

The fecal and serum samples of the patient group were collected twice during hospitalization, including one during the subacute phase at the time of enrollment and the other one during the convalescent phase after 4 weeks of rehabilitation treatment. The samples of the control group were collected from healthy volunteers during routine physical examination.

#### 2.2.4 Sequencing and data preprocessing

Bacterial DNA was extracted, and the 16S rRNA gene was amplified by PCR. The PCR amplicons were sequenced on the PacBio sequencing platform using the single‐molecule real‐time sequencing (SMRT Cell) method. The original off‐machine subreads were corrected to obtain Circular Consensus Sequencing (CCS) sequences (SMRT Link, version 8.0). Then, the Lima (v1.7.0) software was used to identify CCS sequences of different samples through barcode sequences. Chimeras were removed (UCHIME, version 8.1) to obtain high‐quality CCS sequences.

### 2.3 Bioinformatics analyses

Sequences were clustered at a similarity level of 97% using USEARCH (version 10.0), and OUTs (operational taxonomic units) were filtered with a threshold of 0.005% for all sequences. Each OTU represented a taxonomic level, namely kingdom, phylum, class, order, family, genus and species. QIIME2 software was used to analyze Alpha diversity and Beta diversity. Alpha diversity reflects the richness and diversity of microbial species using indices such as Chao1, Ace, Shannon, and Simpson. Beta diversity, which was evaluated with principal coordinate analysis (PCoA) based on the Weighted Unifrac algorithm and the Unweighted Unifrac algorithm, analyzed the difference in the composition and structure of the gut microbiota. Linear discriminant analysis (LDA) effect size (LEfSe) was used to find taxa with significant differences (LDA > 2.5). The Spearman correlation (heatmap) between the microbiota and environmental factors was analyzed and mapped by RDA (redundancy analysis)/CCA (canonical correspondence analysis) in the R language vegan (v2.3) package.

### 2.4 Statistical analysis

Analysis was performed by using SPSS software version 23.0. All data were verified by Shapiro–Wilk test for normality of distribution. If the data conformed to normal distribution, the unpaired *t* test was used for independent samples, and the paired *t* test was used for paired samples. If the data did not conform to the normal distribution, the Wilcoxon rank‐sum test was used for independent samples, and the Wilcoxon signed‐rank test was used for paired samples. Categorical variables were presented as frequencies (percentages), and were tested with chi‐square test. Continuous variables were presented as mean ± SD or medians with interquartile range. *P* < 0.05 was considered statistically significant.

## 3 RESULTS

### 3.1 Demographic and clinical data

Thirty one participants were included in this study with 17 in the healthy control group and 14 in the patient group. The patient group included 12 patients with ischemic stroke and 2 patients with hemorrhagic stroke. There was no statistical difference in gender and age between the two groups, as shown in Table 1. The serum indices, BMI and WHR of the HC group, SS group, and CS group were pairwise compared, and the results showed that the BMI, HDL‐C, LDL‐C and TC were significantly different between the HC group and SS group, as shown in Table 2; the BMI, HDL‐C, LDL‐C and TC were significantly different between the HC group and CS group, as shown in Table 3; the BMI, WHR, RBC, PLT, FMA, BI, MMSE were significantly different between the SS group and CS group, as shown in Table 4.

**TABLE 1:** Comparison of demographic data between healthy control group and patient group.

	Healthy control group (HC, *n* = 17)	Patient group (*n* = 14)	Between‐group comparisons
Age (years)	58.41 ± 9.19	64.57 ± 11.71	*P* > 0.05^a^
Time since onset (days)	12.36 ± 1.65	–	–
Sex			
Female (%)	8 (47.1%)	4 (28.6%)	*P* > 0.05^b^
Male (%)	9 (52.9%)	10 (71.4%)	
Type of stroke	–	Ischemic stroke (*n* = 12)	
	–	Hemorrhagic stroke (*n* = 2)	

*Note*: Data were presented as mean ± SD or *n* (%).

^a^ Unpaired *t* test.

^b^ Chi‐square test.

**TABLE 2:** Results with statistical difference between HC group and SS group.

	HC group (*n* = 17)	SS group (*n* = 14)	Between‐group comparisons
BMI (kg/m^2^)	23.16 ± 1.72	24.73 ± 1.33	*P* ≤ 0.01^a^
HDL‐C (mmol/L)	1.24 ± 0.29	0.91 ± 0.16	*P* ≤ 0.01^a^
LDL‐C (mmol/L)	2.52 ± 0.72	1.93 ± 0.90	*P* ﹤ 0.05^a^
TC (mmol/L)	4.41 (3.77–5.07)	3.35 (2.71–4.07)	*P* ≤ 0.01^b^

*Note*: Data were presented as mean ± SD or median (IQR).

Abbreviations: BMI, body mass index; HC, healthy control; HDL‐C‐, high‐density lipoprotein cholesterol; LDL‐C, low‐density lipoprotein cholesterol; SS, subacute stroke; TC, total cholesterol.

^a^ Unpaired *t* test.

^b^ Wilcoxon rank‐sum test.

**TABLE 3:** Results with statistical difference between HC group and CS group.

	HC group (*n* = 17)	CS group (*n* = 14)	Between‐group comparisons
BMI (kg/m^2^)	23.16 ± 1.72	24.32 ± 1.08	*P* < 0.05^a^
HDL‐C (mmol/L)	1.24 ± 0.29	0.94 ± 0.16	*P* ≤ 0.01^a^
LDL‐C (mmol/L)	2.52 ± 0.72	1.63 ± 0.59	*P* ≤ 0.01^a^
TC (mmol/L)	4.49 ± 0.80	3.18 ± 0.79	*P* ≤ 0.01^a^

*Note*: Data were presented as mean ± SD.

Abbreviations: BMI, body mass index; CS, convalescent stroke; HC, healthy control; HDL‐C, high‐density lipoprotein cholesterol; LDL‐C, low‐density lipoprotein cholesterol; TC, total cholesterol.

^a^ Unpaired *t* test.

**TABLE 4:** Results with statistical difference between SS group and CS group.

	SS group (*n* = 17)	CS group (*n* = 14)	Within‐group comparisons
BMI (kg/m^2^)	24.73 ± 1.33	24.32 ± 1.08	*P* ≤ 0.01^a^
WHR	0.886 ± 0.067	0.867 ± 0.056	*P* ≤ 0.01^a^
PLT (10^9^/L)	226.50 ± 62.16	200.79 ± 37.15	*P* ﹤ 0.05^a^
RBC (10^12^/L)	4.87 (4.11–5.09)	4.67 (4.08–4.87)	*P* ﹤ 0.05^b^
MMSE	15.43 ± 2.71	23.14 ± 2.45	*P* ≤ 0.01^a^
BI	30.00 (25.00–47.50)	70.00 (57.50–82.50)	*P* ≤ 0.01^b^
FMA	22.50 (16.25–44.25)	56.50 (42.25–88.75)	*P* ≤ 0.01^b^

*Note*: Data were presented as mean ± SD or median (IQR).

Abbreviations: BI, Barthel index; BMI, body mass index; CS, convalescent stroke; FMA, Fugl‐Meyer Assessment; MMSE, Mini‐mental State Examination; PLT, platelet count; RBC, red blood cells; SS, subacute stroke; WHR, waist‐hip ratio.

^a^ Paired *t* test.

^b^ Wilcoxon signed‐rank test.

### 3.2 Results of gut microbiota analysis

**FIGURE 1:**
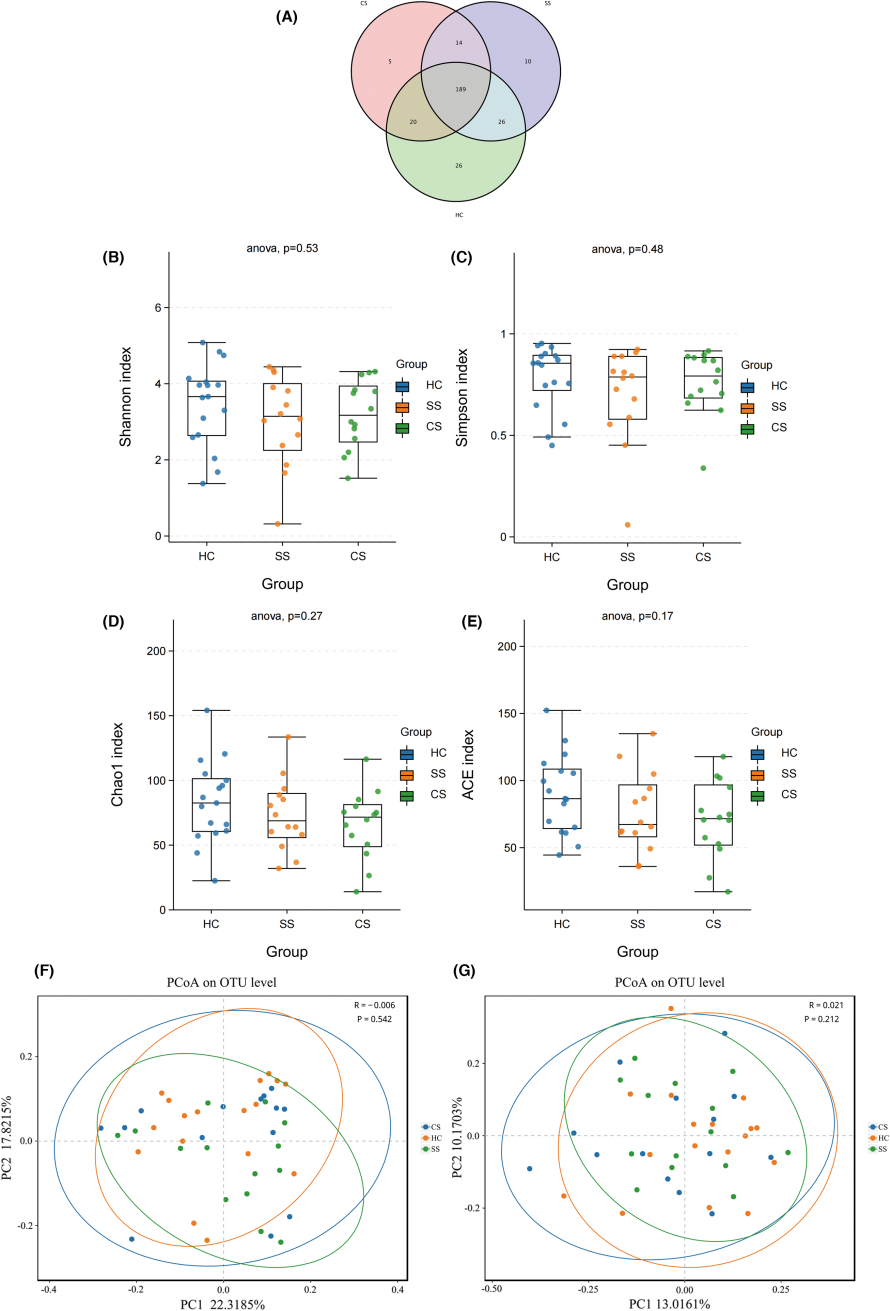
The Venn diagram showed the number of common and unique characteristic OTUs among the three groups of samples, and intuitively showed the coincidence of the characteristics between the samples (A). Alpha diversity analysis showed that there was no statistical difference in species abundance and diversity among the three groups. Shannon and Simpson indexes showed no significant difference in species diversity among healthy control, subacute stroke and convalescent stroke groups (B, C); Chao1 and ACE indexes showed that there was no significant difference in species abundance among the three groups (D, E). PCoA proved that there was no significant difference in the Beta diversity of the gut microbiota between the three groups, according to the Weighted Unifrac distance algorithm (F) and the Unweighted Unifrac distance algorithm (G). PCoA, principal coordinates analysis.

A total of 318,046 CCS sequences identified by Barcode were obtained from 45 samples, including 14 from the SS group, 14 from the CS group, and 17 from the HC group. Each sample generated at least 3306 CCS sequences, with an average of 7068 CCS sequences. The Venn diagram showed a total of 290 OTUs, with a total of 189 common OTUs in all three groups, 26 unique OTUs in the HC group, 10 unique OTUs in the SS group, and 5 unique OTUs in the CS group (Figure 1A).

#### 3.2.1 Comparison of gut microbiota diversity between patient group and healthy control group

Alpha diversity analysis was used to compare the diversity of the gut microbiota. Chao1 and ACE indices measure the abundance, that is, the number of microbial species. Shannon and Simpson indices measure the diversity of species, which are influenced by the abundance of species and evenness of microbial community in the sample.^22^ No statistical difference was found in terms of the overall richness and evenness of gut microbiota community among the three groups, indicating that there was no significant difference in the diversity of gut microbiota between healthy individuals and stroke patients in subacute phase and convalescent phase (Figure 1B–E). Principal coordinate analysis (PCoA) was used to assess the difference among the three groups. PCoA based on weighted Unifrac distance (*R* = −0.006, *P* = 0.542) and unweighted Unifrac distance algorithms (*R* = 0.021, *P* = 0.212) showed a high similarity in terms of microbiota community structure among HC, SS, and CS groups (Figure 1F,G).

#### 3.2.2 The taxonomic distribution of gut microbiota

**FIGURE 2:**
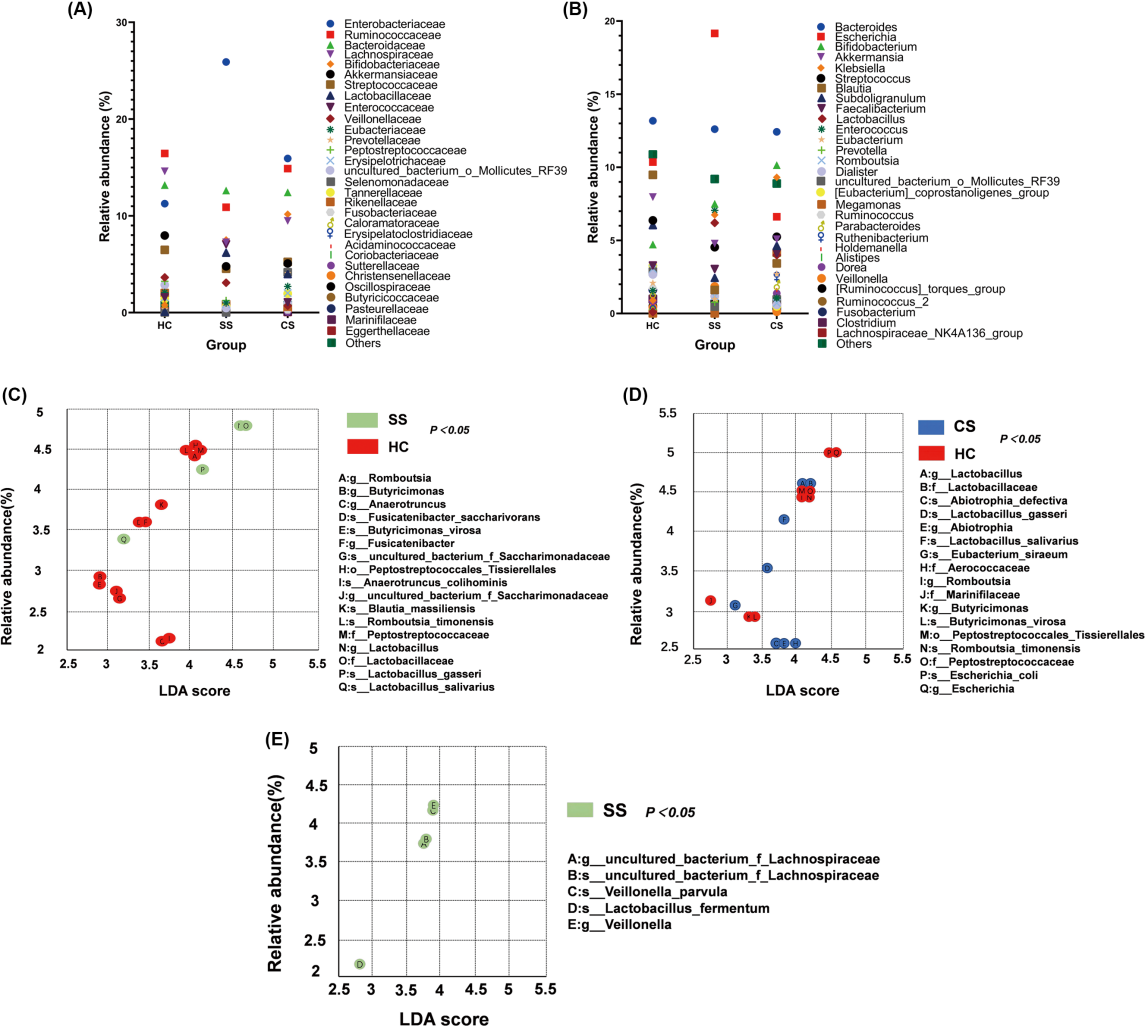
Taxonomic distribution of gut microbiota between healthy control (HC), subacute stroke (SS) and convalescent stroke (CS) groups, at genus level (A), at family level (B). Taxonomic differences of gut microbiota among HC group, SS group and CS group. LEfSe analysis showed the taxa with the most abundant differences between the two groups. Significant difference in gut microbiota between HC group (red) and SS group (green) (C); Significant difference in gut microbiota between CS group (blue) and HC group (red) (D); Significant difference in gut microbiota between SS group (green) and CS group (E); Only LDA score >2.5 and *P* < 0.05 were shown.

The most abundant population at genus level was *Bacteroides*, followed by *Escherichia*, *Bifidobacterium*, and *Akkermansia* (Figure 2A). The most abundant population at family level was *Enterobacteriaceae* in all three groups, followed by *Ruminococcaceae*, *Bacteroidaceae*, and *Lachnospiracea*e. (Figure 2B).

#### 3.2.3 Searching statistically different taxa between different groups by LEfSe

Linear discriminant analysis effect size (LEfSe) showed that the bacterial communities of the SS group had a lower abundance of *Butyricimonas*, *Peptostreptococaceae*, *Romboutsia*, *Anaerotruncus*, *Blautia_massiliensis*, *Fusicatenibacter*, and had a higher abundance of *Lactobacillaceae* compared to the HC group (Figure 2C). Compared with the HC group, the CS group had a lower abundance of *Butyricimonas*, *Peptostreptococcaceae*, *Romboutsia*, *Escherichia*, *Marinifilaceae*, and a higher abundance of *Lactobacillaceae*, *Aerococcaceae*, *Abiotrophia*, *Eubacterium_siraeum* (Figure 2D). Compared with the CS group, the SS group had a higher abundance of *Veillonella* and *Lactobacillus_fermentum*, while there was no community with higher abundance in CS group (Figure 2E).

#### 3.2.4 Heatmap of Correlation analysis between gut microbiota and clinical data

**FIGURE 3:**
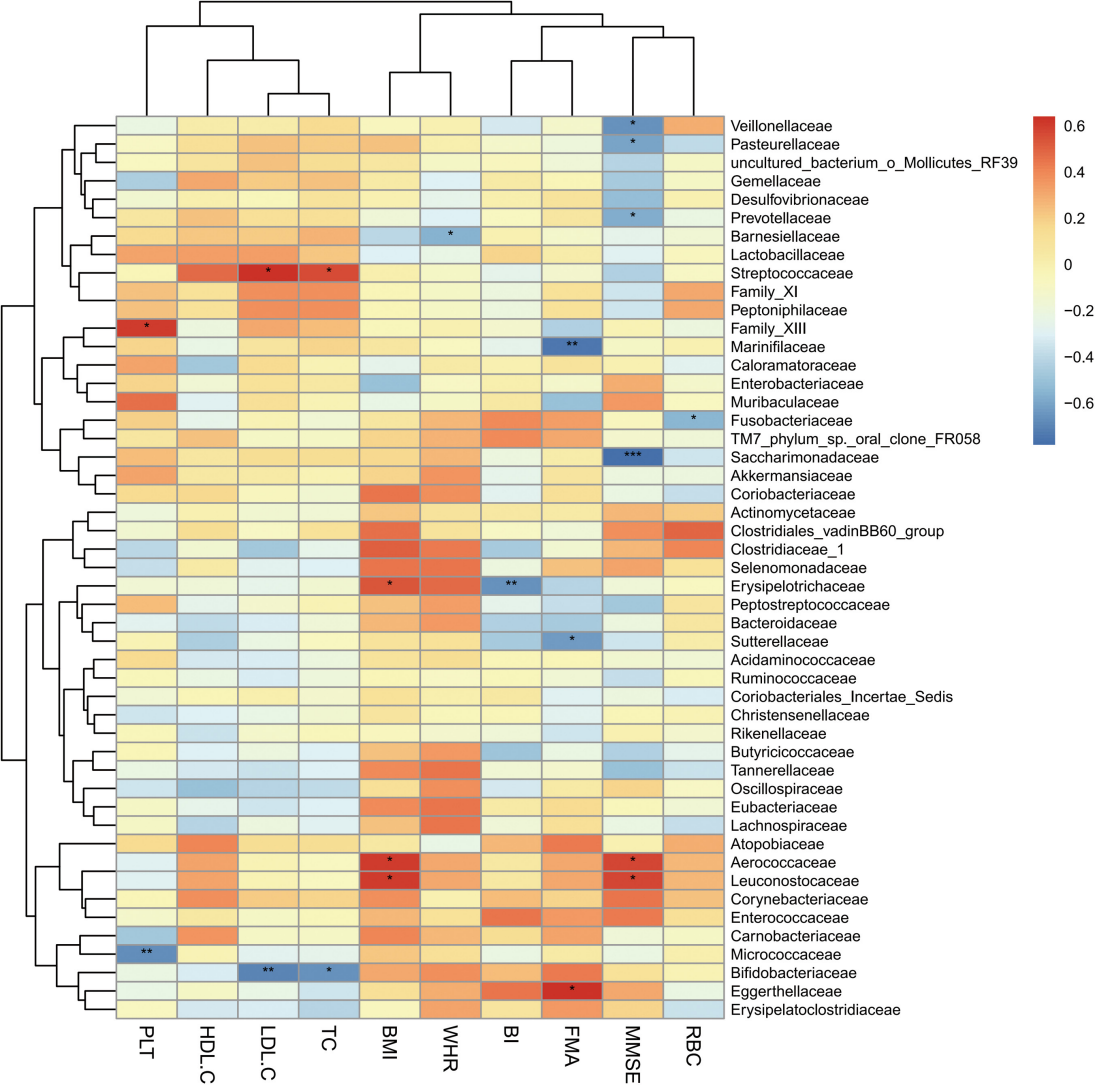
Heatmap of spearman rank correlation analysis between gut microbiota and clinical indexes in subacute stroke group at family level. Red means positive correlation and blue means negative correlation. *P < 0.05, **P < 0.01, ***P < 0.001.

**FIGURE 4:**
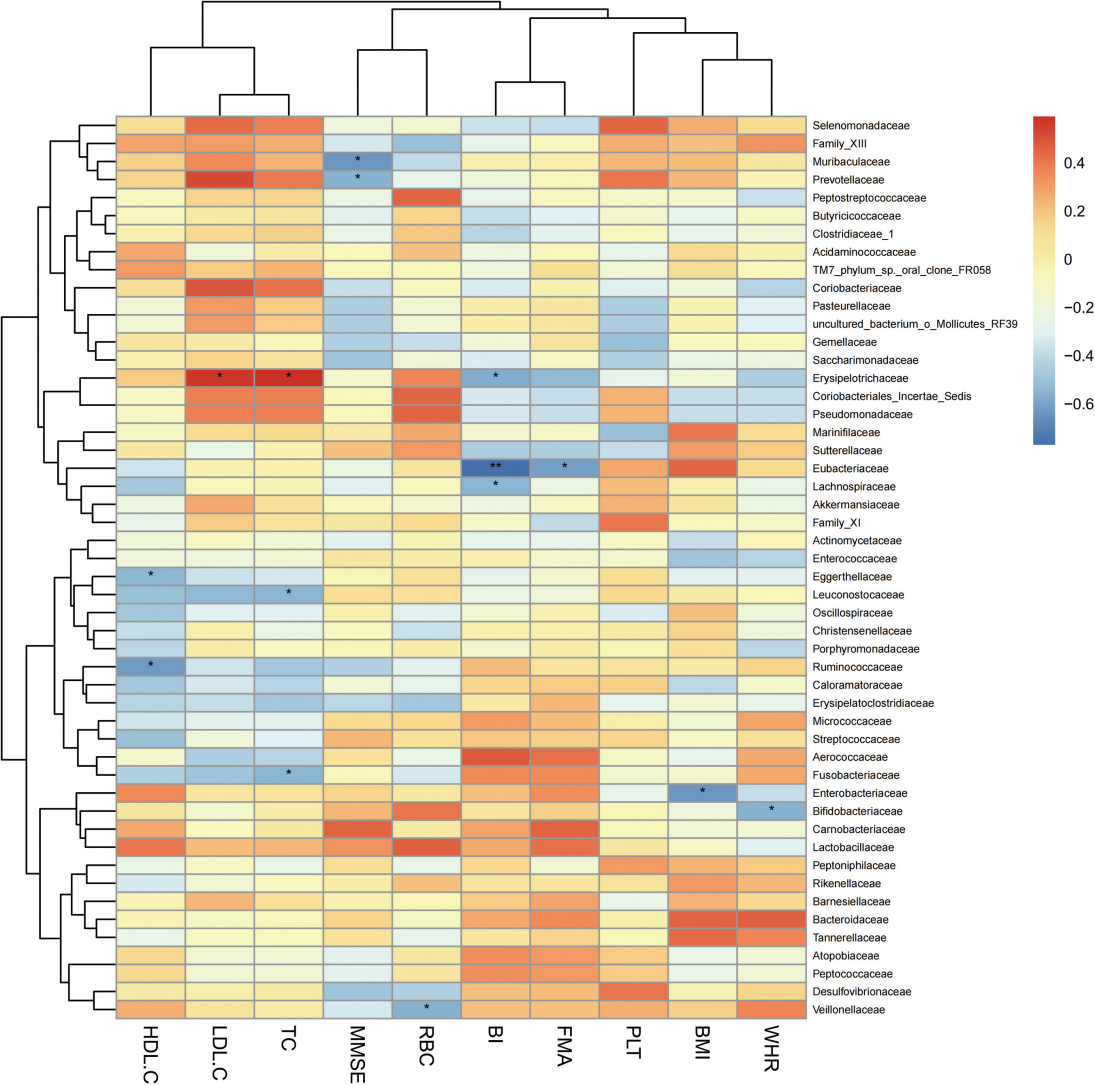
Heatmap of spearman rank correlation analysis between gut microbiota and clinical indexes in convalescent stroke group at family level. Red means positive correlation and blue means negative correlation. *P < 0.05, **P < 0.01, ***P < 0.001.

**FIGURE 5:**
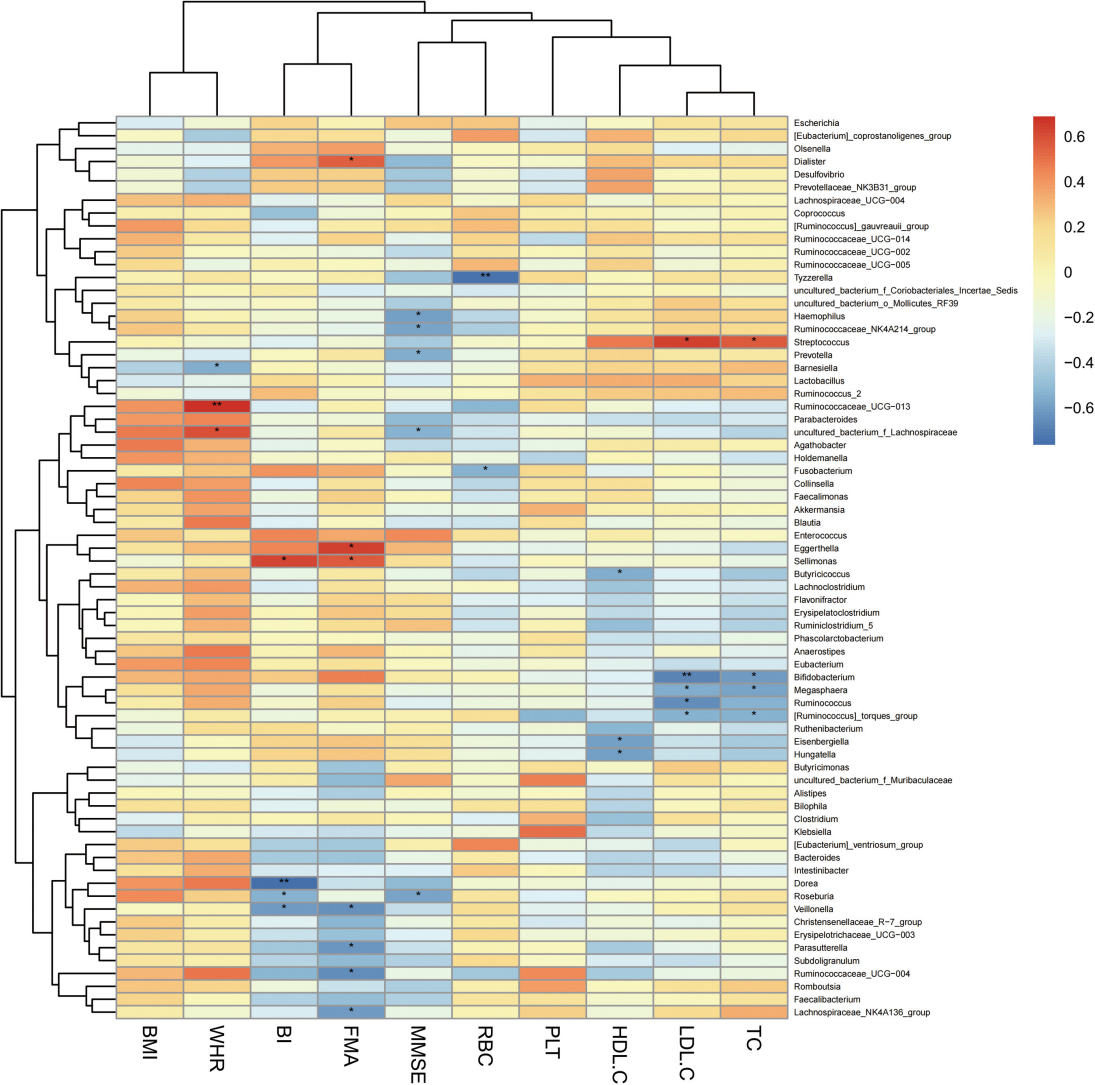
Heatmap of spearman rank correlation analysis between gut microbiota and clinical indexes in subacute stroke group at genus level. Red means positive correlation and blue means negative correlation. *P < 0.05, **P < 0.01, ***P < 0.001.

**FIGURE 6:**
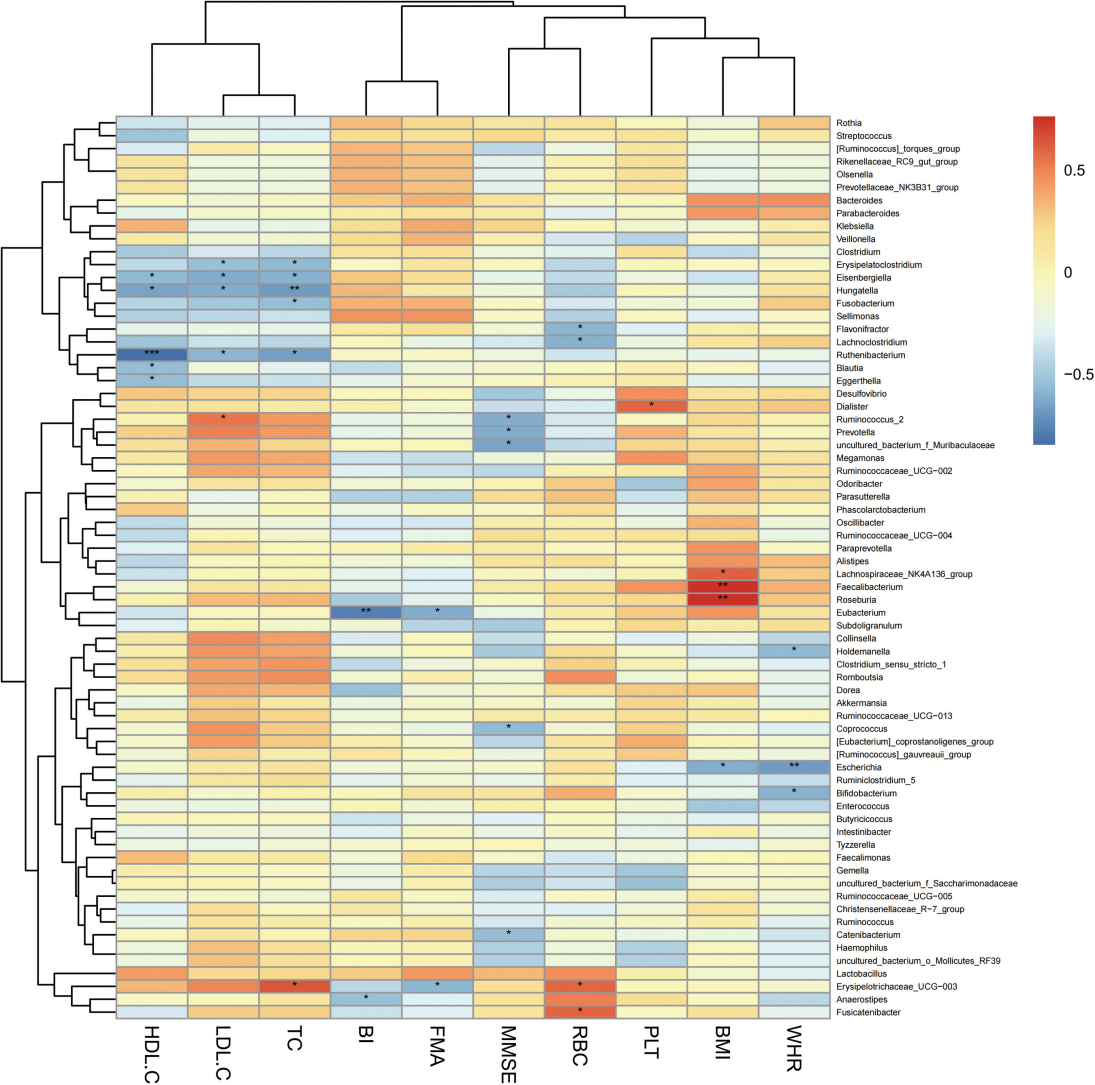
Heatmap of spearman rank correlation analysis between gut microbiota and clinical indexes in convalescent stroke group at genus level. Red means positive correlation and blue means negative correlation. *P < 0.05, **P < 0.01, ***P < 0.001.

Correlation analyses between the most abundant 70 bacterial communities and clinical indices were conducted in the SS group and the CS group (Spearman, *P* < 0.05). *Prevotellaceae* was negatively correlated with MMSE, and *Erysipelotrichaceae* was negatively correlated with BI in both the SS and CS groups at family level (Figures 3 and 4). *Prevotella* was negatively correlated with MMSE, and *Eisenbergilla* and *Hungatella* were negatively correlated with HDL‐C at genus level in both groups (Figures 5 and 6).

## 4 DISCUSSION

The gut microbiota plays a very important role in maintaining human gut health, establishing the gut immune system and protecting the host from pathogen infections. Studies have shown that commensal gut bacteria can affect the host immune system and the disease progression in multiple organs including the brain.^23^, ^24^, ^25^ Our study found that the abundance of bacterial communities in patients with subacute stroke was altered compared with healthy controls, which was consistent with Jia, Xia, and Yamashiro et al's findings which pointed out that gut microbiota would rapidly change and lose its equilibrium after stroke.^26^, ^27^, ^28^ Studies have shown that innate immune cells, including neutrophils, microglia or macrophages, mast cells, innate lymphocytes, and natural killer T‐cells, respond within hours after a stroke, and subsequently produce an adaptive immune response by activating T and B lymphocytes.^29^ Gut dysbiosis can lead to the imbalance of T‐cell subsets, which can aggravate or alleviate ischemic brain injury. Pro‐inflammatory subsets (Th1, Th17) can promote neuroinflammation.^16^ Th1 induces neuroinflammation and activation of microglia by secreting cytokine such as IL‐2, IL‐12 and interferon‐gamma (IFN‐γ).^29^ Th17 activates matrix metalloproteinases and destroys the structure of the blood–brain barrier by producing cytokines such as IL‐17A, IL‐17F and IL‐22.^29^, ^30^ Benakis et al^31^ showed that the disturbance of gut microbiota can reprogram dendritic cells, thereby affecting T lymphocyte differentiation, and enhancing ischemic neuroinflammation by secreting IL‐17. This resulted in increased chemokine production in brain parenchyma and cytotoxic immune cell infiltration, which exacerbated the conditions of stroke patients. Sadler et al^16^ also found that the increase of intestinal segmented filamentous bacteria after stroke can affect the differentiation of T lymphocytes, which reduced the Foxp3/IL‐17 ratio in intestinal lymphoid tissue and expanded pro‐inflammatory Thl7 cell, thus aggravating ischemic brain injury. Xu et al^18^ explored the dynamics of gut microbiota disorder after stroke and its relationship with stroke prognosis through two clinical cohort studies, and also concluded that the gut dysbiosis in the acute stage of stroke was an independent risk factor for poor recovery of stroke patients. These results indicated that the imbalance of gut microbiota affected the degree of brain damage. In order to further explore the changes of gut microbiota after stroke, we compared the CS group with the HC group and found that the number of bacterial communities with lower abundance reduced, whereas the communities with higher abundance increased in the convalescent CS group. We speculated that the gut microbiota was gradually restored during the recovery of post‐stroke patients.

*Butyricimonas*, *Peptostreptococcaceae* and *Romboutsia* were the bacterial communities whose abundance were significantly lower than that of healthy people in the two phases. *Butyricimonas* is a butyrate‐producing species, and butyrate is a short‐chain fatty acid that induces colonic regulatory T cells^32^ which can regulate host physiology, energy metabolism, and immune function. Therefore, it plays an important role in maintaining the integrity of intestinal barrier and inhibiting the production of pro‐inflammatory cytokines^33^; the reduction of butyrate destroys the gut barrier function and promotes inflammation.^34^ Moreover, Chen et al^35^ have found that butyric acid supplementation does not only reduce the rate of neurological impairment and cerebral infarction but also alleviate cerebral infarction edema, reduce blood lipid level, and reduce the risk of thrombosis. It is therefore believed that butyrate‐producing bacteria is one of the therapeutic targets of brain diseases. In this study, *Butyricimonas* in the intestinal tract of stroke patients is significantly reduced compared with that of the healthy controls, which is consistent with the research results of Zeng.^36^ Therefore, we speculate that *Butyricimonas* may affect the course of stroke by affecting the production of butyrate in the intestinal tract. To further explore whether the recovery of stroke patients can be accelerated by supplementing the corresponding bacteria or their products in stroke patients as suggested by Bourassa,^33^ *the difference of Butyricimonas between CS group and SS group was analyzed*. It was found that there was no statistical difference in *Butyricimonas* between the two groups. Nonetheless, it was discovered that the abundance of another butyrate‐producing bacteria *Anaerotruncus* decreased in the SS group compared with the HC group, and the difference was statistically significant; however, there was no significant difference in *Anaerotuncus* between the CS group and the HC group. It can be seen that *Anaerotruncus* was gradually restored during the convalescent phase. Based on this observation, we speculate that butyrate, as a neuroprotective agent, has a positive impact on post‐stroke recovery. The abundance of butyrate‐producing bacteria decreases in the early post‐stroke period, but they are gradually restored to a normal level, which may be one of the factors that contribute to stroke recovery.

Furthermore, some researchers discovered that the decrease of butyrate‐producing bacteria was accompanied by the increase of lactate‐producing bacteria, and lactate was fermented into butyrate to compensate for the lost butyrate‐producing bacteria.^37^ In this study, the abundance of *Lactobacillus*, which produced lactate that could be fermented into butyrate, increased significantly in both the subacute phase and the convalescent phase. *Lactobacillus* is often considered a beneficial gut bacterium that has protective effects on neurological function after ischemia.^38^ Zeng et al^36^ found that the abundance of *Lactobacillus* in the gut of people at high risk of stroke increased while the abundance of butyrate‐producing bacteria and the fecal butyrate concentration decreased, which suggested that *Lactobacillus* has begun to protect the human body as beneficial bacteria before the occurrence of stroke. In order to determine whether *Lactobacillus* is beneficial to nerve recovery, animal experiments were conducted and revealed that intraduodenal injection of *Lactobacillus johnsonii* led to increased gastric vagus nerve activity and decreased renal sympathetic nerve activity in rats, confirming the effect of gut microbiota on nerve functions.^39^ Subsequently, Bravo et al^40^ found that long‐term treatment with probiotic *Lactobacillus rhamnosus* on mice could alter γ‐ aminobutyric acid mRNA expression in specific brain regions, thereby reducing anxiety and depression‐related behaviors via the interference of vagus nerve. Recent clinical studies have found that *Lactobacillus* supplementation can improve the cognitive function and mood of stroke patients. However, others reported that *Lactobacillus* supplementation did not improve the cognitive function.^41^, ^42^ We believe that it is highly likely that *Lactobacillus* is a beneficial bacterium for stroke recovery based on the findings of the current study, but additional study is required to determine the influence and therapeutic effect of *Lactobacillus* on stroke patients in the future.

We found that the BMI of HC group was significantly lower than that of SS group and the CS group, and the BMI and waist‐hip ratio of the CS group were significantly lower than those of the SS group. BMI is an indicator of the degree of obesity, and epidemiological data shows that overweight or obesity is an independent risk factor for stroke.^43^, ^44^ Justyna et al^45^ conducted a study on the correlation between BMI and rehabilitation outcomes after stroke and found that BMI gradually normalized after 5 weeks of rehabilitation treatment, and better functional efficiency was obtained; the positive effect of rehabilitation treatment lasted for 3 months. The authors believed that normalizing BMI may help reduce the risk of complications such as cardiovascular disease and stroke. The results of our study were consistent with Justyna.^45^ Studies conducted in animal models and clinical trials showed that stroke triggered a metabolic shift into the catabolic mode, leading to muscle loss and significant weight loss, and it was suggested that such weight changes may be related to differences in gut microbiota.^46^, ^47^, ^48^ In our study, correlation analysis between the BMI and gut microbiota was conducted, and it was found that the related communities were *Erysipelotrichaceae*, *Aerococcaceae* and *Leuconostocaceae* (positive correlation) in the subacute phase, and the related communities was *Enterobacteriaceae* (negative correlation) in the convalescent phase, confirming the correlation between gut microbiota and body weight after stroke. In addition, our study found that the HDL‐C of the patient group was lower than that of the healthy group while there was no statistical difference in the HDL‐C value between the SS group and CS group. The authors speculated that this result may be related to the fact that the lipid‐lowering drug intervention after stroke had a greater effect on the blood lipid, cholesterol and low‐density lipoprotein, but had little effect on HDL‐C. A prospective cohort study of 267,500 Chinese people in China found that the risk of stroke may increase when HDL‐C was below 50 mg/dL.^49^ Curb et al^50^ also found that high HDL‐C levels were significantly associated with reduced stroke risk. The current study found that *Eisenbergilla* and *Hungatella* were negatively correlated with HDL‐C in stroke patients. *Eisenbergiella* is more abundant in people with high‐fat or low‐fiber diet intake, and its level significantly increases in obese people. Such an increase is positively correlated with weight gain, implying a possible negative correlation between this bacterium and HDL‐C.^51^, ^52^ This paper is the first to discover the correlation between HDL‐C and gut microbiota after stroke. We hope that future research can continue to explore the correlation between HDL‐C and gut microbiota after stroke in order to provide guidance for stroke prevention and treatment.

It was observed that the motor‐related FMA scores, cognition‐related MMSE scores, and ADL‐related BI scores were all improved significantly from subacute phase to convalescent phase after 4 weeks of rehabilitation treatment. Correlation analysis between the scores and gut microbiota of the two phases found that *Prevotellaceae* at family level and *Prevotella* at genus level were negatively correlated with cognitive function score of MMSE. The results of this study were consistent with those of Ling's.^53^ Chen et al^54^ suspected that the increased abundance of *Prevotellaceae* may be related to the inflammatory response after stroke, and Chesnokova et al^55^pointed out that the inflammatory signaling pathway activated by gut microbial products can affect the cognition, learning, memory and other functions of brain. Magnusson et al^56^ also found that the systemic inflammatory response caused by the change of gut microbiota composition can further induce cognitive defects such as the decline of working and spatial memory. To sum up, by considering the prior works mentioned above and our results, we speculate that stroke patients with cognitive impairment may have impaired intestinal barrier in the early stage of stroke or even before stroke, which causes the gut microbiota in the GI tract to leak into the blood stream and other organs, thereby inducing the body's immune response, inflammatory response and neurodegeneration, and eventually affecting the cognitive function after stroke. This study confirmed the correlation between the gut microbiota and cognition. We believe that colonization of gut microbiota has the potential to improve cognitive dysfunction after stroke and is a treatment regimen of cognitive impairment after stroke, but further research and verification are needed in the future.

## 5 CONCLUSION

To sum up, gut microbiota of patients with stroke was maladjusted. In the subacute phase, more bacterial communities showed decreased abundance. With rehabilitation treatment after stroke, gut microbiota was gradually restored to the level of the healthy people. *Lactobacillus* was significantly increased in the gut after stroke, which may compensate for the reduction of butyrate‐producing bacteria, and *Lactobacillus* may be a beneficial bacterium after stroke. Gut microbiota may affect the functional status of stroke by affecting BMI and/or cognitive scores, thereby changing the prognosis of stroke. Gut microbiota transplantation may be one of the targets for future stroke treatment.

The main drawbacks of this study include that the sample size was small, the observation time was relatively short, and the gut microbiota transplantation was not conducted to verify the curative effect of the gut microbiota for stroke. Many researchers have observed the improvement of stroke outcomes through gut microbiota interventions, including methods such as fecal transplantation, beneficial bacterial supplementation, dietary management, and drug treatment, and found that it can significantly reduce the level of brain inflammation and neurological impairment after stroke.^35^, ^57^, ^58^, ^59^ In future studies, we hope to expand the sample size, extend the treatment observation time, observe gut‐derived metabolites and their functions (especially stroke‐related metabolites), and explore methods that may help to tailor the supplements to establish healthy microbiota, so as to further promote better post‐stroke recovery outcomes.

## CONFLICT OF INTEREST STATEMENT

Authors declare no conflict of interest.

## Data Availability

The data that support the findings of this study are available from the corresponding author upon reasonable request.
